# Metabolome and transcriptome analysis on muscle of sporadic inclusion body myositis

**DOI:** 10.1002/acn3.51657

**Published:** 2022-09-15

**Authors:** Ayuka Murakami, Seiya Noda, Tomoyuki Kazuta, Satoko Hirano, Seigo Kimura, Hirotaka Nakanishi, Koji Matsuo, Koyo Tsujikawa, Madoka Iida, Haruki Koike, Kazuma Sakamoto, Yuichiro Hara, Satoshi Kuru, Kenji Kadomatsu, Teppei Shimamura, Tomoo Ogi, Masahisa Katsuno

**Affiliations:** ^1^ Department of Neurology Nagoya University Graduate School of Medicine Nagoya Japan; ^2^ Department of Neurology National Hospital Organization Suzuka Hospital Suzuka Japan; ^3^ Department of Neurology Yokkaichi Municipal Hospital Yokkaichi Japan; ^4^ Department of Neurology Kariya Toyota General Hospital Kariya Japan; ^5^ Department of Biochemistry Nagoya University Graduate School of Medicine Nagoya Japan; ^6^ Institute for Glyco‐Core Research (iGCORE), Nagoya University Nagoya Japan; ^7^ Department of Genetics Research Institute of Environmental Medicine (RLeM), Nagoya University Nagoya Japan; ^8^ Department of Human Genetics and Molecular Biology Nagoya University Graduate School of Medicine Nagoya Japan; ^9^ Division of Systems Biology Nagoya University Graduate School of Medicine Nagoya Japan; ^10^ Department of Clinical Research Education Nagoya University Graduate School of Medicine Nagoya Japan

## Abstract

**Objective:**

Sporadic inclusion body myositis (sIBM) is the most common acquired myopathy in patients older than 50 years of age. sIBM is hardly responds to any immunosuppressing theraphies, and its pathophysiology remains elusive. This study aims to explore pathogenic pathways underlying sIBM and identify novel therapeutic targets using metabolomic and transcriptomic analyses.

**Methods:**

In this retrospective observational study, we analyzed biopsied muscle samples from 14 sIBM patients and six non‐diseased subjects to identify metabolic profiles. Frozen muscle samples were used to measure metabolites with cation and anion modes of capillary electrophoresis time of flight mass spectrometry. We validated the metabolic pathway altered in muscles of sIBM patients through RNA sequencing and histopathological studies.

**Results:**

A total of 198 metabolites were identified. Metabolomic and transcriptomic analyses identified specific metabolite changes in sIBM muscle samples. The pathways of histamine biosynthesis and certain glycosaminoglycan biosynthesis were upregulated in sIBM patients, whereas those of carnitine metabolism and creatine metabolism were downregulated. Histopathological examination showed infiltration of mast cells and deposition of chondroitin sulfate in skeletal muscle samples, supporting the results of metabolomic and transcriptomic analyses.

**Interpretation:**

We identified alterations of several metabolic pathways in muscle samples of sIBM patients. These results suggest that mast cells, chondroitin sulfate biosynthesis, carnitine, and creatine play roles in sIBM pathophysiology.

## Introduction

Sporadic inclusion body myositis (sIBM) is the most common inflammatory myopathy in the elderly,^1^ and the number of patients is gradually increasing worldwide.^2^, ^3^, ^4^ The main symptom of sIBM is progressive weakness of the quadriceps and finger and wrist flexors, leading to loss of grip strength, gait disturbance, and eventual shortening of healthy life expectancy.^1^, ^5^, ^6^ Dysphagia due to bulbar palsy is reported in about half of patients, although respiratory failure is uncommon.^7^ Immunosuppressing therapies, including steroids, often fail to suppress progression, and no curative therapies have been identified for sIBM.^5^

The pathogenesis of sIBM is thought to have two mechanisms: inflammation and degeneration. Inflammatory mechanisms are supported by infiltration of CD8‐positive cells in histopathology and anti‐cytosolic 5′‐nucleotidase 1A (cN1A) autoantibodies in the sera of a certain population of sIBM patients.^8^ Degenerative mechanisms are indicated by protein deposition, such as amyloid, TAR DNA‐binding protein 43, and tau,^9^ which are strongly associated with age‐related neurodegenerative disorders, including Alzheimer's disease and amyotrophic lateral sclerosis (ALS), in muscle fibers. Myopathy with histopathological features similar to sIBM is often found in families with ALS and frontotemporal dementia, of which several causative genes, including *VCP*, have been identified,^10^ further inferring the degenerative nature of sIBM. Additionally, genetic analysis reveals variants in the gene encoding FYVE and coiled‐coil domain‐containing protein 1 (FYCO1), which regulates autophagosome transport, in sIBM patients.^11^ The precise disease mechanism of sIBM, however, remains largely unknown.

Metabolomics is a method for identifying changes in metabolic pathways by identifying small molecules in a biological system, but few studies have explored the metabolomics of muscular diseases. A study of the metabolomic analysis of various myopathies revealed alterations in several metabolic pathways, including transsulfuration, creatine, and niacinamide, in the blood of sIBM patients,^12^ although such changes have not been validated in skeletal muscle.

The present study aimed to elucidate the metabolic profiles of sIBM via metabolomic and transcriptomic analyses of biopsied muscle specimens of sIBM patients and identify novel therapeutic targets of this devastating age‐related myopathy.

## Materials and Methods

### Patients

To identify the metabolic profile of sIBM muscle, we analyzed muscle samples biopsied from 14 sIBM patients (10 men and four women) and age‐ and sex‐matched six healthy subjects. In an attempt to perform RNA sequencing, we also analyzed muscle samples biopsied from 12 sIBM patients (eight men and four women) and age‐ and sex‐matched five non‐diseased subjects. Of those, seven sIBM and five non‐diseased subjects underwent both metabolomic and transcriptomic analyses. All sIBM patients were diagnosed as clinicopathological defined IBM according to the European Neuromuscular Centre IBM research diagnostic criteria 2011.^6^ Patients were not on immunotherapy for sIBM, except one patient who had previously received oral steroid therapy. Non‐diseased samples were collected from patients with minor muscular symptoms, such as myalgia, of whom histological studies showed no evidence of myopathy. All samples were collected from Japanese. This study was conducted according to the Declaration of Helsinki, the Ethics Guidelines for Human Genome/Gene Analysis Research, and the Ethical Guidelines for Medical and Health Research Involving Human Subjects endorsed by the Japanese government. This study was approved by the Ethics Review Committee of Nagoya University Graduate School of Medicine (No. 2016‐0157), and all participants provided written informed consent before participation.

### Muscle biopsies

We performed an open muscle biopsy for diagnostic purposes, as previously described.^13^ Frozen 10 μm sections were examined via routine histological procedures for diagnosis. Frozen muscle samples were stored at −80°C until used. Patients did not fast before the biopsy, which was performed 3–4 h after meals for all subjects.

### Metabolome extraction

Metabolite extraction was conducted at Human Metabolome Technologies (HMT), Japan. For capillary electrophoresis time of flight mass spectrometry (CE‐TOFMS) analysis, approximately 30 mg of frozen muscle tissue was plunged into 750 μL of 50% acetonitrile/Milli‐Q water containing internal standards (Solution ID: 304‐1002, HMT) at 0°C to deactivate enzymes. Tissues were homogenized 10 times at 3500 rpm for 60 sec using a tissue homogenizer, and then, the homogenate was centrifuged at 2300*g* and 4°C for 5 min. Subsequently, 400 μL of the upper aqueous layer was centrifugally filtered through a Millipore 5 kDa cutoff filter at 9100*g* and 4°C for 120 min to remove proteins. The filtrate was centrifugally concentrated and resuspended in 50 μL of Milli‐Q water for CE‐TOFMS analysis.

### Metabolomic analysis

Metabolome measurements were performed through a facility service at HMT. CE‐TOFMS was performed using an Agilent CE Capillary Electrophoresis System equipped with an Agilent 6210 Time of Flight mass spectrometer, Agilent 1100 isocratic HPLC pump, Agilent G1603A CE‐MS adapter kit, and Agilent G1607A CE‐ESI‐MS sprayer kit (Agilent Technologies, Waldbronn, Germany). The systems were controlled by Agilent G2201AA ChemStation software version B.03.01 for CE (Agilent Technologies, Waldbronn, Germany). Metabolites were analyzed using a fused silica capillary (50 μm i.d. × 80 cm total length), with commercial electrophoresis buffer (Solution ID: H3301‐1001 for cation analysis and H3302‐1021 for anion analysis, HMT) as the electrolyte. Samples were injected at a pressure of 50 mbar for 10 sec (approximately 10 nL) for cation analysis and 25 sec (approximately 25 nL) for anion analysis. The spectrometer was scanned from *m*/*z* 50 to 1000. Other conditions were performed as described previously.^14^, ^15^

Peaks were extracted using automatic integration software MasterHands (Keio University, Tsuruoka, Japan) to obtain peak information, including *m*/*z*, migration time for CE‐TOFMS measurement (MT), and peak area.^16^ Signal peaks corresponding to isotopomers, adductions, and other productions of known metabolites were excluded and the remaining peaks were annotated with putative metabolites from the HMT metabolite database on the basis of MTs and *m*/*z* values determined via TOFMS. The tolerance range for peak annotation was configured at ±0.5 min for MT and ±10 ppm for *m*/*z*. Furthermore, peak areas were normalized against internal standards; then, resultant relative area values were further normalized according to sample amount. Multiple possible metabolites were noted if several annotations were assigned to a single peak.

### RNA extraction and sequencing processing

We extracted RNA from frozen biopsied muscle tissues of 12 sIBM patients and five non‐diseased subjects using the miRNeasy Mini Kit (Qiagen). Total RNA was quantified using a 2100 Bioanalyzer with an RNA 6000 Pico Kit (Agilent Technologies). Libraries were prepared using the Illumina TruSeq Stranded Total RNA Library Prep according to the manufacturer's protocol (Illumina) and then sequenced with Illumina HiSeq 2500 (PE‐150 nt).

### Quantification and analysis of RNA‐seq data

Adapter sequence trimming and low‐quality (quality value <20) base removal were processed with Trim Galore v. 0.5.0 (https://www.bioinformatics.babraham.ac.uk/projects/trim_galore/). The quality of sequence reads was assessed using FastQC v. 0.11.8 (https://www.bioinformatics.babraham.ac.uk/projects/fastqc/) before and after trimming. Processed reads were mapped on human genome version hg19 using HISAT2 v. 2.1.0^17^ by applying strand specificity information, followed by removing reads mapped to rDNA regions with BEDTools v. 2.25.0.^18^ Individual gene expressions were quantified with StringTie v. 1.3.5^19^ using hg19 gene annotation of the UCSC Genome Browser. Counts per million and transcripts per million values were computed with StringTie and edgeR v. 3.24.3^20^ implemented in R v. 3.5.1, respectively.

RNA‐seq‐based expression data were analyzed using integrated Differential Expression and Pathway analysis (iDEP).^21^ To compare two groups (sIBM vs. non‐diseased), we performed a pairwise comparison using log‐transformed transcripts per million values on the limma package (false discovery rate (FDR) cutoff: 0.1, fold change cutoff: 2.0). For pathway analysis to explore pathways changing in the skeletal muscle of sIBM patients, we used Parametric Gene Set Enrichment Analysis (PGSEA, FDR cutoff: 0.2) as a method and Kyoto Encyclopedia of Genes and Genomes (KEGG) as gene sets.

### Mast cell staining and cell count

To determine mast cells, sections of muscle samples were fixed and stained with toluidine blue according to a previously reported procedure.^22^ We selected the suitable area for evaluation and counted mast cells in at least four areas per sample using ImageJ software v. 1.51 (NIH, Bethesda, Maryland, USA).

### Immunohistochemical analysis

Immunohistochemical studies were performed as previously described.^23^ Briefly, serial 10‐μm‐thick sections were fixed in ice‐cold acetone and incubated with normal horse serum or normal goat serum for 60 min, followed by incubation with primary antibodies. After washing with phosphate‐buffered saline, sections were incubated with biotinylated horse antimouse immunoglobulin G or biotinylated horse antimouse immunoglobulin M (Vector Laboratories) for 60 min. Next, sections were washed extensively and exposed to avidin–biotin complex (Vector Laboratories) for 60 min and were then covered with diaminobenzidine for 10 min. Primary antibodies were mouse anti–HLA‐ABC (555551, Becton‐Dickinson, 1:4000), mouse anti‐CD8 (M7103, Dako, 1:150), mouse anti‐p62 (ab56416, Abcam, 1:300), mouse anti‐chondroitin sulfate proteoglycan (C8035, Sigma‐Aldrich, 1:2000), mouse anti‐keratan sulfate (PRPG‐BG‐M01, Cosmo Bio Co, 1:30), and mouse anti‐heparan sulfate (H1890, US Biological, 1:50).

### Statistical analysis

Targeted metabolomics data were analyzed using MetaboAnalyst 3.0.^24^ Multivariate analysis was performed using partial least squares discriminant analysis (PLS‐DA). The values of variable importance in projection (VIP) indicated the importance of metabolites for the PLS‐DA models. Data were log‐transformed and autoscaled before statistical analysis. We performed metabolic pathway analysis using reactome pathway analysis (https://www.reactome.org/) on the 20 metabolites with high VIP scores. RNA‐seq data were assessed using Prism 8 (GraphPad Software). Values were presented as mean ± standard deviation. Student's t‐test or Welch's t‐test was used for comparing two groups.

## Results

### Metabolomic analysis and transcriptomic analysis of sIBM skeletal muscle

**Figure 1:**
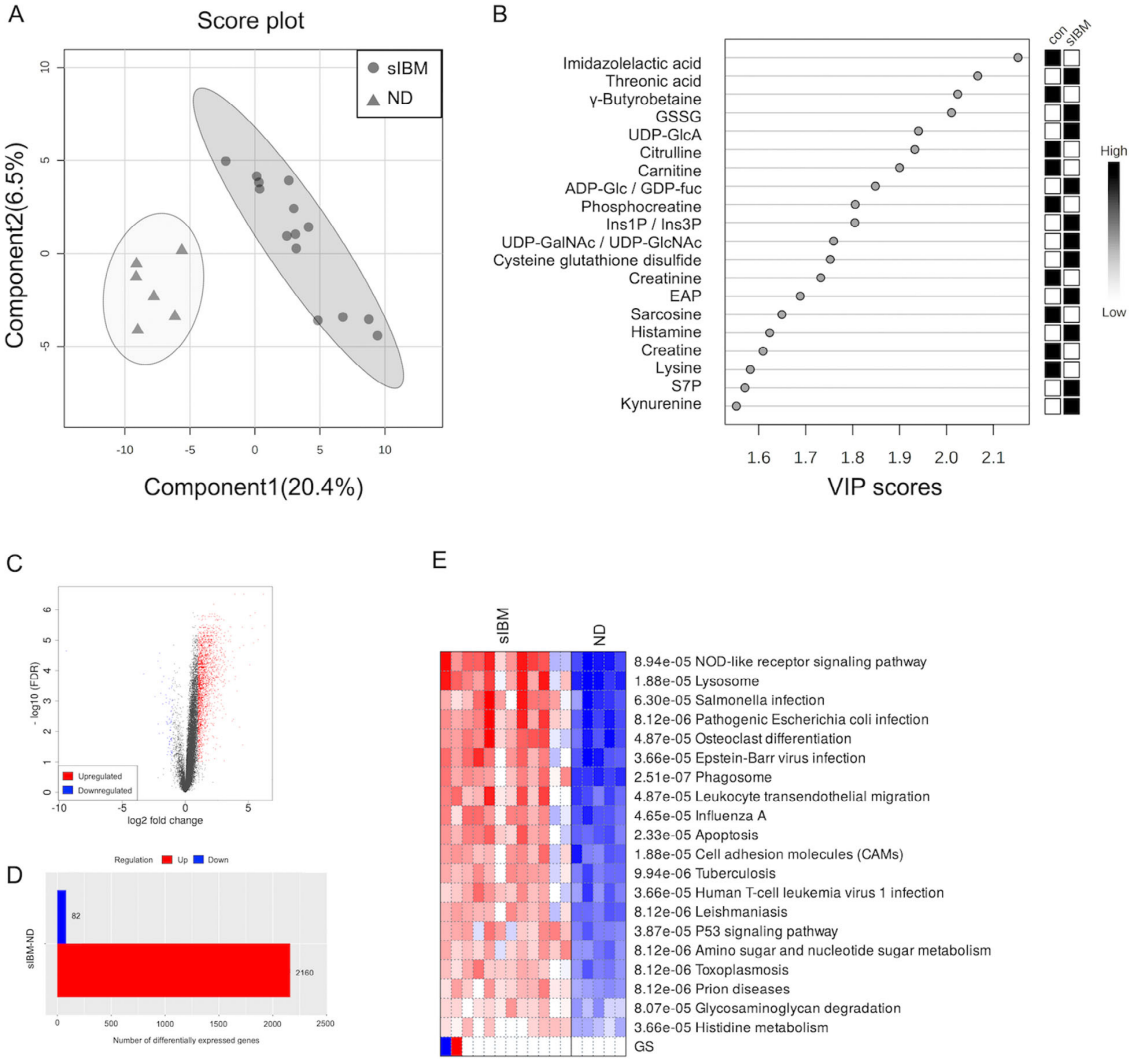
Clustering of metabolome and RNA‐seq data in sIBM patients and non‐diseased subjects. (A) PLS‐DA plots of metabolites. (B) VIP score plots of top 20 metabolites. (C) Volcano plots of RNA‐seq data. (D) Number of differentially expressed genes using the limma package. (E) Enrichment pathway analysis of RNA‐seq data with the Kyoto Encyclopedia of Genes and Genomes (KEGG). GSSG, Glutathione disulfide_divalent; UDP‐GlcA, uridine diphosphate‐glucuronic acid; ADP‐Glc, adenosine diphosphate‐glucose; GDP‐fuc, guanosine diphosphate–fucose; Ins1P, myoinositol 1‐phosphate; Ins3P, myoinositol 3‐phosphate; ND, non‐diseased; UDP‐GalNAc, uridine diphosphate N‐acetylgalactosamine; UDP‐GlcNAc, uridine diphosphate N‐acetylglucosamine; EAP, ethanolamine phosphate; S7P, sedoheptulose 7‐phosphate.

Table 1 provides the clinical background for the subjects. For both metabolomic and transcriptomic analyses, sIBM and non‐diseased groups were matched for age and sex. Based on *m*/*z* values and migration time, a total of 198 metabolites were identified in CE‐TOFMS analysis. PLS‐DA showed that non‐diseased subjects and sIBM groups were clearly separated (Fig. 1A). The top 20 metabolites with high VIP scores (Fig. 1B), which discriminate between sIBM and non‐diseased subjects, were associated with the histamine biosynthesis pathway (imidazolelactic acid and histamine), nucleotide sugar associated with glycosaminoglycan metabolism [uridine diphosphate‐glucuronic acid (UDP‐GlcA), adenosine diphosphate‐glucose (ADP‐Glc), and guanosine diphosphate–fucose (GDP‐fuc), uridine diphosphate N‐acetylgalactosamine (UDP‐GalNAc), and uridine diphosphate N‐acetylglucosamine (UDP‐GlcNAc)], carnitine metabolism (γ‐butyrobetaine and carnitine) and creatine metabolism (phosphocreatine and creatine), suggesting that these pathways were dysregulated in sIBM. Pathway analysis revealed several altered pathways (Table 2) in sIBM skeletal muscle comprising those associated with amino acids, glycosaminoglycan, nucleotide sugars, SLC‐mediated transmembrane transport, organic cation transport, creatine, histamine, and carnitine. The results of metabolomic analysis thus indicate that histamine biosynthesis pathways, glycosaminoglycan metabolism, amino acid metabolism, nucleotide sugars metabolism, carnitine metabolism, and creatine metabolism were altered in sIBM skeletal muscle.

**Table 1:** Demographic characteristics of sIBM patients and controls.

	Characteristics	sIBM patients	Non‐diseased	*p*‐value
Metabolome analysis	No. of subjects	14	6	
	Male	10 (71%)	4 (67%)	0.84
	Age at biopsy (years)	69.9 ± 5.9	67.7 ± 5.9	0.55
	Age at onset (years)	65.4 ± 5.3		
	Duration (months)	53.7 ± 31.1		
	CK (U/L)	537 ± 391	136 ± 130	<0.05
	Biopsy site (biceps brachialis: quadriceps)	9:5	3:3	0.57
Transcriptome analysis	No. of subjects	12	5	
	Male	8 (67%)	4 (80%)	0.60
	Age at biopsy (years)	67.6 ± 6.7	71.0 ± 5.7	0.37
	Age at onset (years)	63.1 ± 6.3		
	Duration (months)	55.5 ± 31.7		
	CK (U/L)	728 ± 413	128 ± 142	<0.001
	Biopsy site (biceps brachialis: quadriceps)	9:3	3:2	0.80

Seven sIBM and 5 control subjects underwent both metabolomic and transcriptomic analyses. Values are presented as the mean ± standard deviation. sIBM, sporadic inclusion body myositis; CK, creatine kinase.

**Table 2:** Pathway analysis of metabolites in sIBM skeletal muscle.

	Pathway name	Entities found	Entities *p* value	Entities FDR
R‐HSA‐71291	Metabolism of amino acids and derivatives	11	5.92E‐06	0.00101853
R‐HSA‐3595177	Defective CHSY1 causes TPBS	2	1.64E‐04	0.01325292
R‐HSA‐727802	Transport of nucleotide sugars	3	2.33E‐04	0.01325292
R‐HSA‐71288	Creatine metabolism	3	3.53E‐04	0.01391215
R‐HSA‐425407	SLC‐mediated transmembrane transport	7	4.09E‐04	0.01391215
R‐HSA‐2022870	Chondroitin sulfate biosynthesis	2	0.00100627	0.02817555
R‐HSA‐549127	Organic cation transport	3	0.00172504	0.04140104
R‐HSA‐382551	Transport of small molecules	7	0.00228729	0.04803315
R‐HSA‐549132	Organic cation/anion/zwitterion transport	3	0.00338684	0.06434988
R‐HSA‐425366	Transport of bile salts and organic acids, metal ions, and amine compounds	4	0.00473589	0.08051015
R‐HSA‐5619102	SLC transporter disorders	4	0.00568048	0.08520727
R‐HSA‐9033807	ABO blood group biosynthesis	2	0.0075081	0.08729434
R‐HSA‐3560782	Diseases associated with glycosaminoglycan metabolism	2	0.0075081	0.08729434
R‐HSA‐390650	Histamine receptors	1	0.00933546	0.08729434
R‐HSA‐5619078	Defective SLC35C1 causes congenital disorder of glycosylation 2C (CDG2C)	1	0.00933546	0.08729434
R‐HSA‐5083636	Defective GALNT12 causes colorectal cancer 1 (CRCS1)	1	0.00933546	0.08729434
R‐HSA‐5083625	Defective GALNT3 causes familial hyperphosphatemic tumoral calcinosis (HFTC)	1	0.00933546	0.08729434
R‐HSA‐71262	Carnitine synthesis	2	0.00969937	0.08729434
R‐HSA‐5619115	Disorders of transmembrane transporters	4	0.01111772	0.09294427
R‐HSA‐5173105	O‐linked glycosylation	2	0.01214175	0.09294427

As for transcriptomic analysis, we identified 2,160 upregulated genes and 82 downregulated genes (Fig. 1C and D). Among the top 20 pathways from running PGSEA on KEGG gene sets, the majority of the differentially expressed genes were associated with infection pathways, suggesting an inflammatory aspect of the pathogenesis of sIBM (Fig. 1E). The pathways associated with amino sugar and nucleotide sugar metabolism, glycosaminoglycan degradation, and histidine metabolism were also altered as in metabolomic analysis (Fig. 1E).

### Histamine biosynthesis pathway in the muscle of sIBM patients

**Figure 2:**
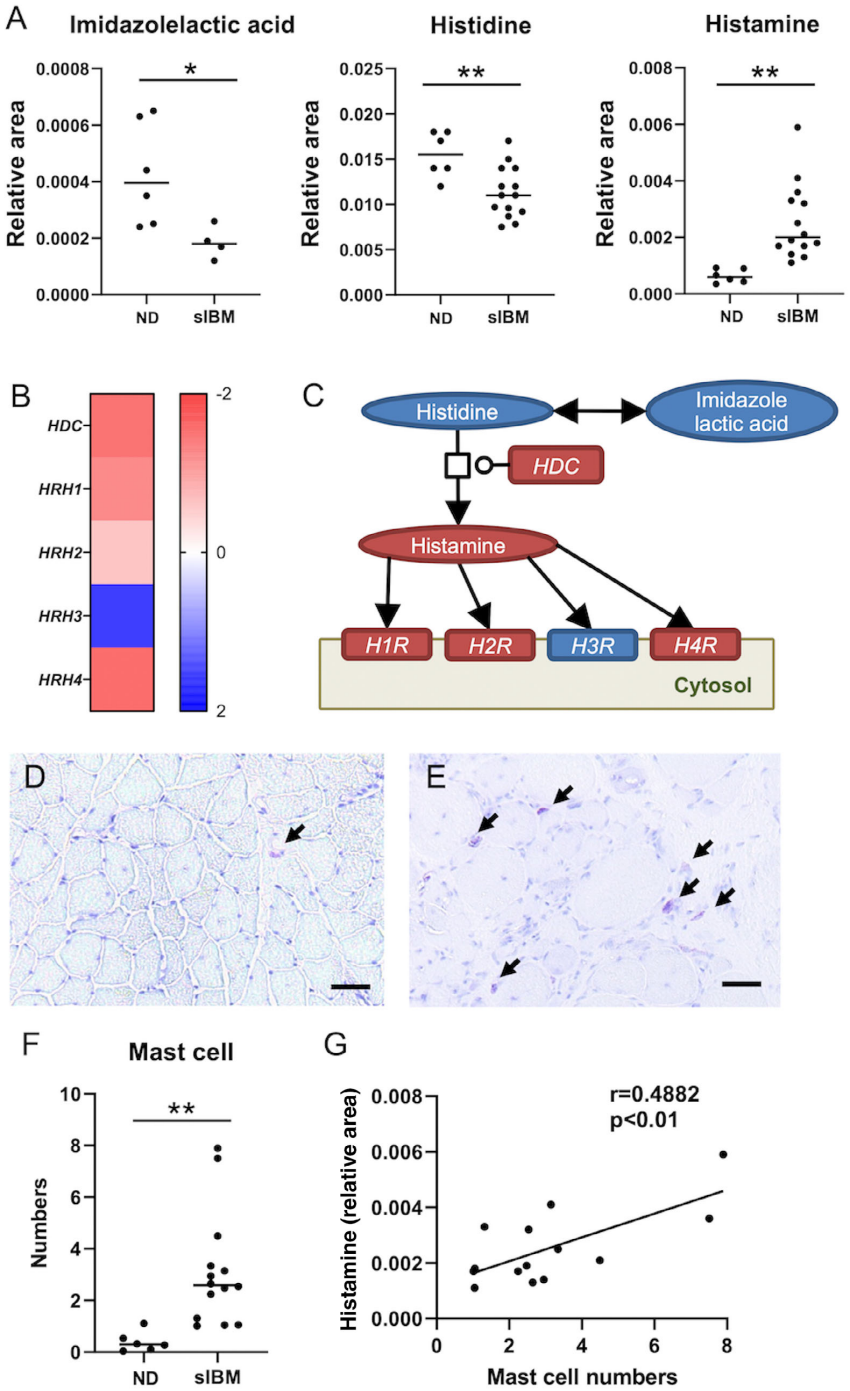
Histamine biosynthesis pathway in sIBM skeletal muscle. (A) Levels of imidazolelactic acid, histidine, and histamine in muscle samples of non‐diseased subjects and sIBM patients. (B) Heat map of log fold changes (FC) for histamine biosynthesis‐related transcripts. Color bar represents logFC value. (C) Histamine biosynthesis pathway in sIBM skeletal muscle. Red circles and rectangles represent upregulated metabolites and transcripts, respectively. Blue circles and a rectangle represent downregulated metabolites and transcripts, respectively. (D and E) Toluidine blue staining of muscle samples from a representative non‐diseased subject (D) and sIBM subjects (E). Black arrows indicate mast cells. (F) Number of mast cells in muscle samples of non‐diseased subjects and sIBM patients. (G) Correlation between the number of mast cells and histamine levels in muscle samples of sIBM patients. Scale bar = 50 μm. *p < 0.05, **p < 0.01. The *p* values were calculated using Welch's t test (A) and Student's t test (F). HDC = histidine decarboxylase; H1R = histamine receptor type 1; H2R = histamine receptor type 2; H3R = histamine receptor type 3; H4R = histamine receptor type 4; ND = non‐diseased.

For the histamine biosynthesis pathway, imidazolactic acid and histidine levels were lower in sIBM skeletal muscle than in non‐diseased subjects, whereas histamine levels, which are produced from histidine by histidine decarboxylase (HDC), increased more in sIBM skeletal muscle than in non‐diseased subjects (Fig. 2A). In support of these findings, mRNA expression levels of *HDC* increased in sIBM skeletal muscle (Fig. 2B). The mRNA expression levels of *histamine receptors type 1*, *2*, and *4* also increased in sIBM skeletal muscle (Fig. 2B). Collectively, these results indicate that histamine biosynthesis via HDC and histamine release from mast cells were enhanced and those histamine receptors were possibly upregulated by evaluated histamine levels (Fig. 2C). To validate this view, we further performed mast cell immunostaining. Although mast cells were identified as toluidine blue‐positive cells in both non‐diseased muscle (Fig. 2D) and sIBM muscle (Fig. 2E), their numbers within muscle specimens were significantly higher in patients with sIBM than in non‐diseased subjects (*p* < 0.01; Fig. 2F). Mast cell numbers also correlated with histamine levels (Fig. 2G).

### Amino sugar, nucleotide sugar, and glycosaminoglycan metabolism in sIBM patient muscles

**Figure 3:**
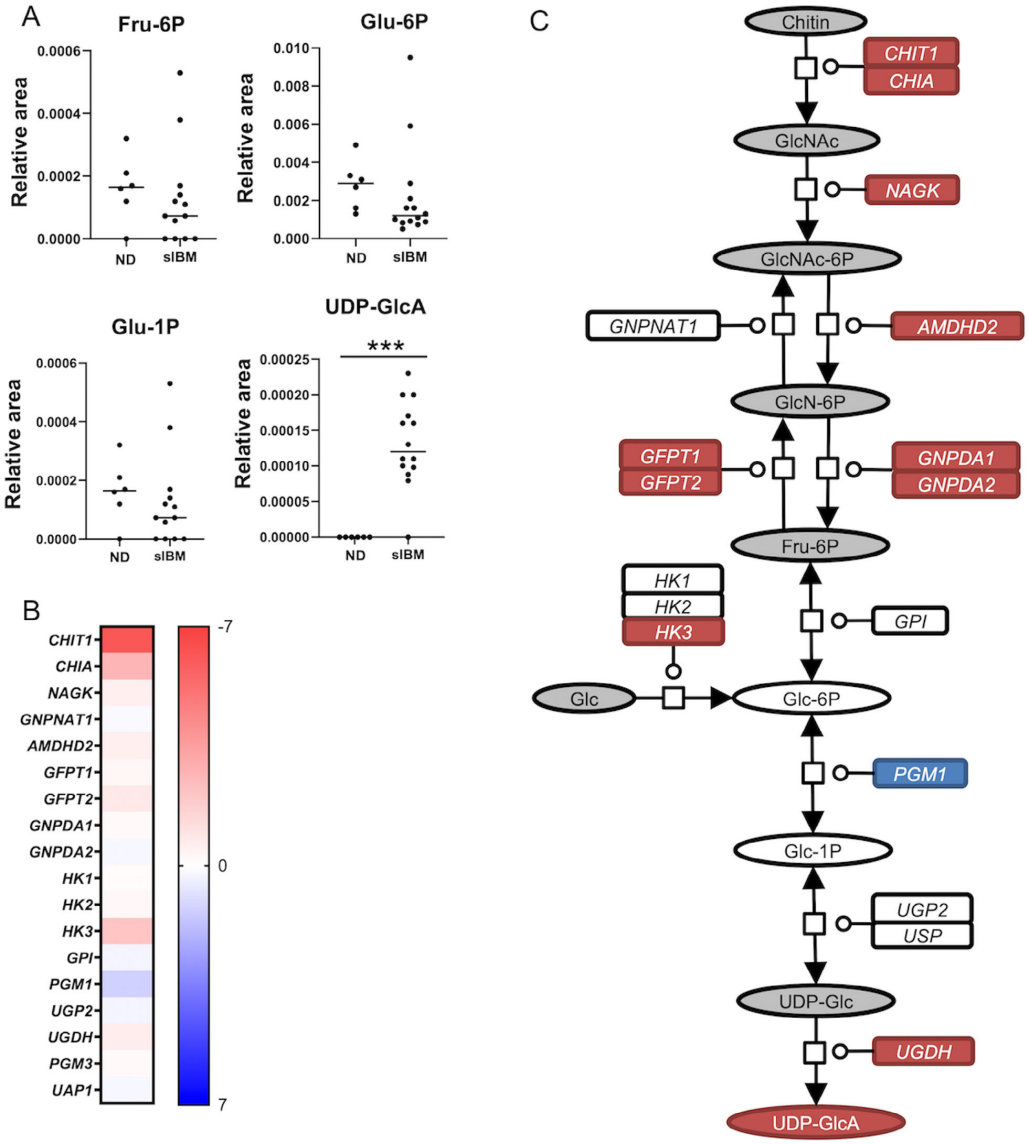
Amino sugar and nucleotide sugar metabolism in sIBM skeletal muscle. (A) Levels of Fru‐6P, Glu‐6P, Glu‐1P, and UDP‐GlcA in muscle samples of non‐diseased subjects and sIBM patients. (B) Heat map of log fold changes (FC) for amino sugar and nucleotide sugar‐related transcripts. Color bar represents logFC value. (C) Amino sugar and nucleotide sugar metabolism in sIBM skeletal muscle. Red circles and rectangles represent upregulated metabolites and transcripts, respectively. Blue circles and rectangles represent downregulated metabolites and transcripts, respectively. Gray circles and rectangle represent unmeasured metabolites and transcripts, respectively. *p < 0.05, **p < 0.01, ***p < 0.001. The *p* values were calculated using Welch's t test. Fru‐6P = fructose 6‐phosphate; Glu‐6P = glucose 6‐phosphate; Glu‐1P = glucose 1‐phosphate; GlcNAc‐1P = N‐acetylglucosamine 1‐phosphate; ND = non‐diseased; UDP‐GlcA = uridine diphosphate‐glucuronic acid.

**Figure 4:**
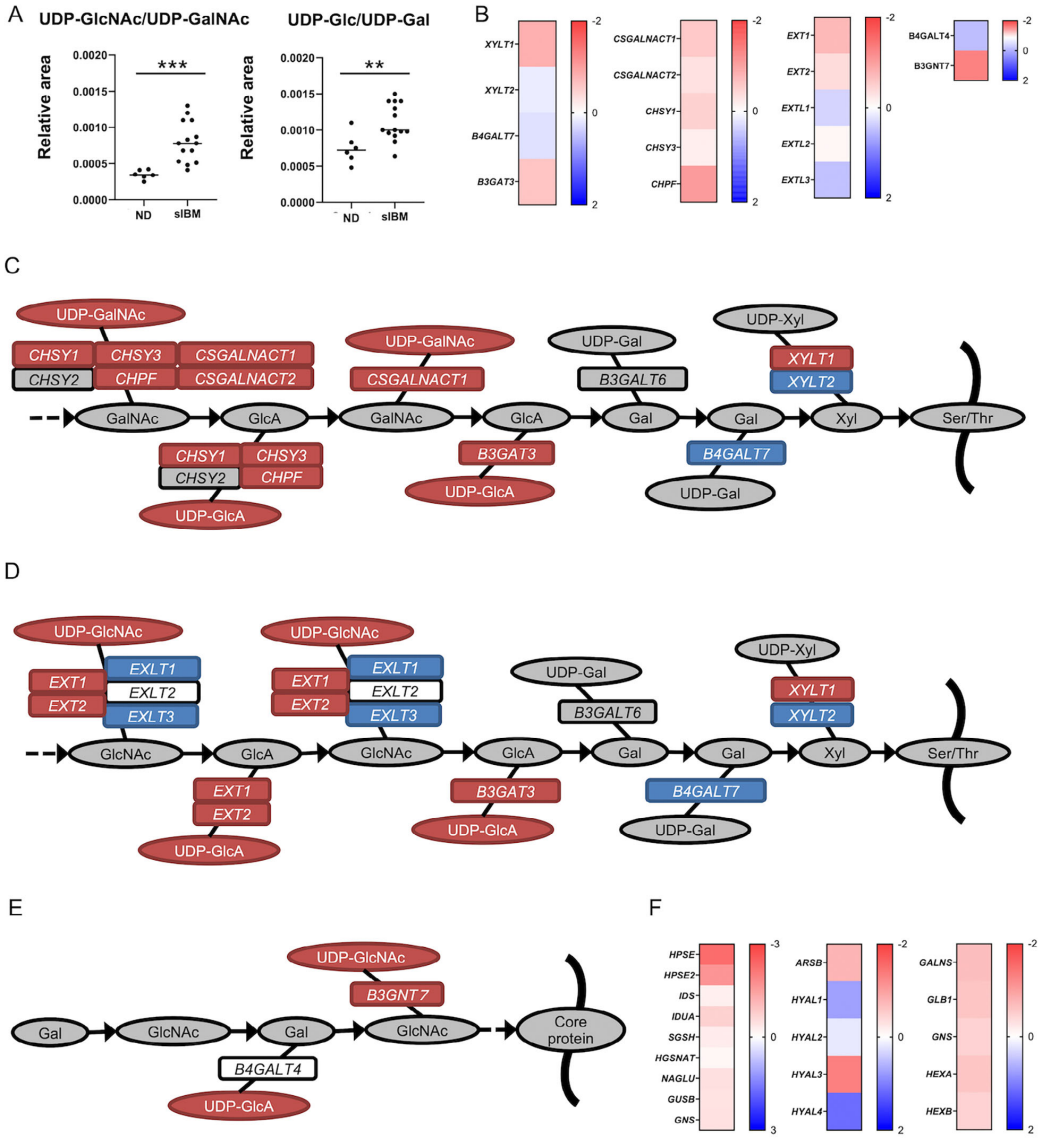
Glycosaminoglycan biosynthesis and degradation in sIBM skeletal muscle. (A) Levels of UDP‐GlcNAc or UDP‐GalNAc and UDP‐Glc or UDP‐Gal in muscle sample of non‐diseased subjects and sIBM patients. (B) Heat map of log fold changes (FC) for glycosaminoglycan biosynthesis‐related transcripts. Color bar represents logFC value. (C–E) Glycosaminoglycan biosynthesis pathways [heparan sulfate (C), chondroitin sulfate (D), and keratan sulfate (E)] in sIBM skeletal muscle. Color bar represents logFC value. Red circles and rectangles represent upregulated metabolites and transcripts, respectively. Blue circles and rectangles represent downregulated metabolites and transcripts, respectively. Gray circles and rectangles represent unmeasured metabolites and transcripts, respectively. (F) Heat map of log fold changes (FC) for glycosaminoglycan degradation‐related transcripts. *p < 0.05, **p < 0.01, ***p < 0.001. The *p* values were calculated using Welch's t test. ND = non‐diseased; UDP‐GalNAc = uridine diphosphate N‐acetylgalactosamine; UDP‐GlcNAc = uridine diphosphate N‐acetylglucosamine; UDP‐Glc = uridine diphosphate‐glucose; UDP‐Gal = uridine diphosphate‐galactose.

**Figure 5:**
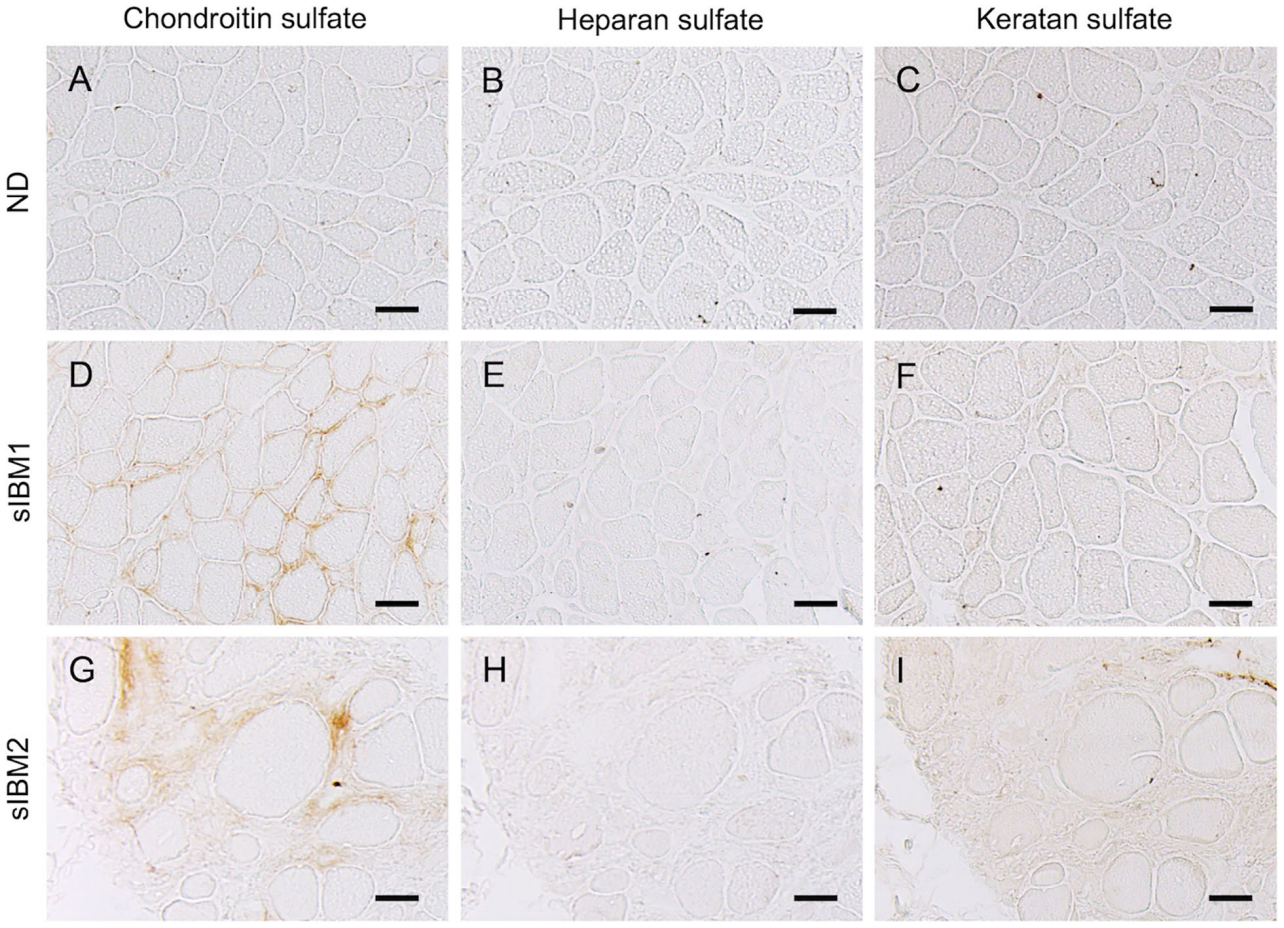
Glycosaminoglycan immunohistochemistry of biopsied muscle samples. Representative images of immunohistochemistry of muscle specimens from a non‐diseased subject (A–C) and sIBM patients (D–I). Stains include chondroitin sulfate (A, D, G), heparan sulfate (B, E, H), and keratan sulfate (C, F, I). Scale bar = 50 μm.

Within the amino sugar and nucleotide sugar metabolism pathways, UDP‐GlcA levels, part of nucleotide sugar, were higher in sIBM, whereas other metabolites were not (Fig. 3A). The mRNA levels of genes associated with biosynthesis of N‐acetylglucosamine (GlcNAc) and UDP‐GlcA were upregulated (Fig. 3B and C). Nucleotide sugar levels, UDP‐GlcNAc/UDP‐GalNAc and UDP‐Glc/UDP‐Gal, were also higher in sIBM skeletal muscle than in non‐diseased subjects (Fig. 4A). The mRNA levels of several genes associated with glycosaminoglycan biosynthesis were also upregulated (Fig. 4B). These results indicate that the biosynthesis of glycosaminoglycan, particularly chondroitin sulfate, was amplified in the skeletal muscle of sIBM subjects (Fig. 4C–E). For the glycosaminoglycan degradation pathway, the mRNA levels of almost all genes associated with heparan sulfate degradation and keratan sulfate degradation were upregulated, whereas for *HYAL1*, *HYAL2*, and *HYAL4*, genes associated with chondroitin sulfate degradation were downregulated (Fig. 4F). To validate these findings, we performed further immunohistochemical analysis (Fig. 5). To reflect omics data, connective tissues and sarcolemma in sIBM muscle specimens were strongly immunostained by anti‐chondroitin sulfate antibody compared with non‐diseased specimens. Neither heparan sulfate nor keratan sulfate immunoreactivity was observed in non‐diseased or sIBM muscle specimens.

### Carnitine and creatine metabolism in muscles of sIBM patients

**Figure 6:**
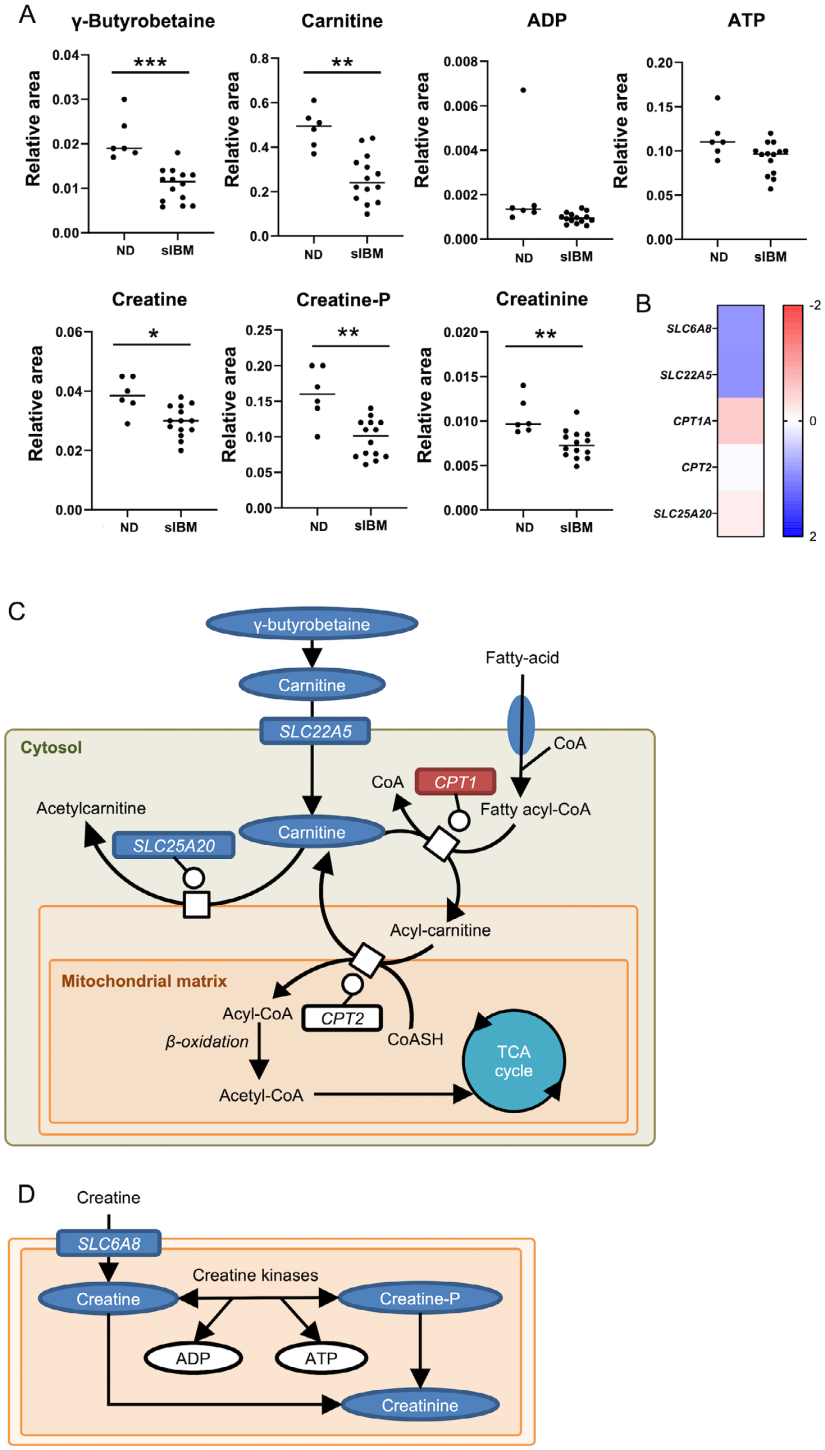
Carnitine and creatine metabolism in sIBM skeletal muscle. (A) Levels of γ‐butyrobetaine, carnitine, ADP, ATP, creatine, phosphocreatine (creatine‐P), and creatinine in muscle samples of non‐diseased subjects and sIBM patients. (B) Heat map of log fold changes (FC) for carnitine and creatine metabolism‐related transcripts. Color bar represents logFC value. (C–D) Carnitine metabolism (C) and creatine metabolism (D) in sIBM skeletal muscle. Red circles and rectangles represent upregulated metabolites and transcripts, respectively. Blue circles and rectangles represent downregulated metabolites and transcripts, respectively. * *p* < 0.05, **p < 0.01, *** *p* < 0.001. The *p* values were calculated using Welch's t test. CoA = Coenzyme A; Creatine‐P = phosphocreatine; ND = non‐diseased.

Carnitine and creatine metabolism have been shown to be altered in blood samples of patients with muscular disorders including sIBM.,^12^, ^25^, ^26^ ^,42^ We found lower levels of γ‐butyrobetaine, carnitine, creatine, phosphocreatine, and creatinine in sIBM skeletal muscle than in non‐diseased subjects (Fig. 6A). mRNA levels of *SLC22A5* (carnitine transporter), *SLC25A20* (carnitine‐acylcarnitine carrier), and *SLC6A8* (creatine transporter) were lower in sIBM skeletal muscle than non‐diseased subjects (Fig. 6B). These results indicate that for carnitine metabolism, the uptake of carnitine in skeletal muscle and the transport of acylcarnitine to the mitochondrial matrix were attenuated in sIBM (Fig. 6C). Regarding creatine metabolism, creatine uptake in skeletal muscle was suppressed in sIBM (Fig. 6D).

## Discussion

In this study, we showed several, altered metabolic pathways in the skeletal muscle of patients with sIBM, which was supported by gene expression analysis. The pathways we identified included histamine biosynthesis, glycosaminoglycan biosynthesis, carnitine metabolism, and creatine metabolism.

Our study revealed upregulation of histamine biosynthesis in metabolome analysis and RNA‐seq combined with mast cell infiltration in sIBM skeletal muscle. Previous studies reveal that histamine performs a dual role in muscle disease. It is hypothesized that histamine is a deteriorative factor in polymyositis (PM)^27^ while being protective in Duchenne muscular dystrophy (DMD) model mice.^28^ The pathogenetic role of histamine has been indicated in a study showing that the number of mast cells increased in the affected muscle of PM patients and that infiltration of CD8‐positive T cells in skeletal muscle is reduced in C protein‐induced myositis developed in mast cell‐deficient mice.^27^ It is well known that invasion of CD8‐positive T cells in myofibrils plays an important role in the pathophysiology of sIBM, as with PM.^1^ Conversely, mast cells accumulate in skeletal muscle because of myofiber membrane disruption^29^ and histamine release from mast cells contributes to vasodilation in skeletal muscle and glucose availability to skeletal muscle following exercise^30^ via histamine H1 and H2 receptor activation.^31^ These findings highlight the beneficial role of histamine in myopathy, particularly for muscle regeneration. Consequently, in DMD model mice, histamine administration improves grip strength and performance.^28^ Combined, histamine may play a detrimental role in inflammation and a protective role in the regeneration of sIBM muscle tissue. Further studies are needed to elucidate the precise role of histamine and mast cells in sIBM.

Within the nucleotide sugar metabolism pathway, we revealed enhanced biosynthesis of some nucleotide sugars, which are components of glycosaminoglycans. We also demonstrated insufficient degradation of chondroitin sulfate in metabolomic and transcriptomic analyses, as well as its deposition in the interstitium of sIBM muscle specimens from histopathological studies. By contrast, neither heparan sulfate nor the keratan sulfate pathways were affected in histopathology. These pathological and transcriptomic findings suggest that excessive chondroitin sulfate is involved in sIBM pathophysiology. Chondroitin sulfate proteoglycan 4 plays a role in both fibrogenic differentiation induced by transforming growth factor‐β (TGF‐β) and adipogenic differentiation from basic fibroblast growth factor in skeletal muscle.^32^ Given that the activation of TGF‐β signaling has been shown in skeletal muscles of sIBM,^33^ chondroitin sulfate may exacerbate the pathogenesis of this disease. Furthermore, chondroitin sulfate plays a key role in muscle regeneration. A temporal decrease in chondroitin sulfate levels is needed for skeletal muscle differentiation and regeneration, whereby an injection of chondroitin sulfate‐degrading enzyme can improve dystrophic pathology in DMD model mice.^34^ These findings suggest that accelerating chondroitin sulfate degradation may be a therapeutic approach to degenerating pathology of sIBM skeletal muscle.

We also found carnitine and creatine deficiency in sIBM skeletal muscle. Both carnitine and creatine play important roles in energy metabolism. Carnitine deficiency in skeletal muscle has been described in various myopathies^35^ but scarcely documented for sIBM. In skeletal muscle, carnitine transports long‐chain fatty acids into mitochondria to promote β‐oxidation.^25^ Several reports also show carnitine supplementation improves lipid metabolism by ameliorating mitochondrial dysfunction through autophagy induction when regulating peroxisome proliferator‐activated receptor γ (PPARγ) expression.^26^, ^36^ Conversely, creatine plays an essential role in energy storage. Creatine is synthesized from glycine, arginine, and S‐adenosylmethionine in the liver and kidney; exported to skeletal muscle; and phosphorylated for short‐term energy storage.^37^ As with carnitine, creatine deficiency in skeletal muscle has also been described in various neuromuscular disease^38^, ^39^ and creatine supplementation therapy improves muscle strength in patients with muscular dystrophy.^40^ A recent study on myopathy metabolomes found the creatine/creatinine ratio significantly increased in sIBM patient blood, indicating low creatine pools.^12^ As shown above, carnitine and creatine pathways appear to be therapeutic targets of sIBM.

The present study has several limitations. First, this was an exploratory study with a limited sample size. Second, since participants did not fast before the biopsy, the effect of metabolic changes due to dietary content cannot be excluded. Furthermore, only Japanese subjects were included in the study, although the effects of ethnicity on sIBM have not been clarified.

In summary, our results indicate that metabolic pathways for histamine, chondroitin sulfate, creatine, and carnitine are altered in the skeletal muscle of sIBM patients and are potential therapeutic targets for this devastating myopathy for which no effective treatment has been available.

## Conflicts of Interest

Nothing to report.

## Author Contributions

A.M., S.N., and M.K. contributed to the conception and design of the study; A.M., S.N., T.K., S.H., S.Ki., H.N., K.M., K.T., M.I., H.K., K.S., and Y.H. contributed to the acquisition and analysis of the data; A.M. and S.N. contributed to drafting the text and preparing the figure; and S.Ku., K.K., T.S., T.O., and M.K. revised the manuscript critically for important intellectual content.
